# Genetically predicted gestational age and birth weight are associated with cardiac and pulmonary vascular remodelling in adulthood

**DOI:** 10.1093/eurjpc/zwad296

**Published:** 2024-05-11

**Authors:** Art Schuermans, Maddalena Ardissino, Victor Nauffal, Shaan Khurshid, James P. Pirruccello, Patrick T. Ellinor, Adam J. Lewandowski, Pradeep Natarajan, Michael C. Honigberg

**Affiliations:** 1 Cardiovascular Disease Initiative and Program in Medical and Population Genetics, Broad Institute of Harvard and MIT, Cambridge, MA, USA; 2 Cardiovascular Research Center and Center for Genomic Medicine, Massachusetts General Hospital, Boston, MA, USA; 3 Department of Cardiovascular Sciences, KU Leuven, Leuven, Belgium; 4 National Heart and Lung Institute, Imperial College London, London, UK; 5 Cardiovascular Epidemiology Unit, Department of Public Health and Primary Care, University of Cambridge, Cambridge, UK; 6 Cardiovascular Division, Brigham and Women’s Hospital, Boston, MA, USA; 7 Demoulas Center for Cardiac Arrhythmias, Massachusetts General Hospital, Boston, MA, USA; 8 Division of Cardiology and Institute for Human Genetics, University of California San Francisco, San Francisco, CA, USA; 9 Oxford Cardiovascular Clinical Research Facility, Division of Cardiovascular Medicine, Radcliffe Department of Medicine, University of Oxford, Oxford, UK; 10 Division of Cardiology, Massachusetts General Hospital, 185 Cambridge St. CPZN 3.187, Boston, 02114 MA, USA

Preterm birth (<37 weeks gestation) and small for gestational age (SGA; <10th percentile) each affect ~10–15% of live births worldwide. Individuals born at an early gestational age (GA) and/or low birth weight (BW) more often develop pulmonary hypertension or heart failure later in life, potentially due to pulmonary vascular and cardiac remodelling from birth to adulthood.^1–3^ However, most evidence linking these factors to cardiopulmonary remodelling stems from observational studies^1–3^ that were potentially affected by sociodemographic confounding. To mitigate the cardiopulmonary impact of early GA and low BW, it is necessary to understand whether each factor contributes causally to cardiovascular remodelling in adulthood. Here, we tested the associations of genetically predicted GA and BW *Z*-score (adjusted for GA) on measures of cardiac and pulmonary arterial (PA) structure and function using Mendelian randomization (MR).

We used two-sample MR to infer causal associations of GA and BW *Z*-score with cardiac and PA structure and function. Mendelian randomization tests whether an exposure–outcome relationship is causal by leveraging the random assortment of genetic variants as instrumental variables. Single-nucleotide variants (SNVs) associated with BW *Z*-score and GA were identified in 84 689 and 26 836 European-ancestry participants, respectively, from the Early Growth Genetics (EGG) Consortium.^4,5^ The EGG Consortium is a large-scale collaboration including >40 genotyped cohorts with data on birth characteristics such as BW (pooled mean: 3.45 ± 0.57 kg) and GA (pooled mean: 39.8 ± 1.8 weeks).^4,5^ Single-nucleotide variants for cardiac and PA structure and function were extracted from genome-wide association studies including up to 43 230 European-ancestry UK Biobank participants who underwent cardiac magnetic resonance (CMR) imaging.^6–9^ Structural measures were indexed to body surface area (using the Mosteller^8^ or Dubois^6,7^ formulas). There was no sample overlap between exposure and outcome cohorts. Since only one SNV reached genome-wide significance (*P* < 5 × 10^−8^) for GA, primary analyses utilized a lenient significance threshold (*P* < 5 × 10^−3^) using the robust adjusted profile score (MR-RAPS) method—a method that accommodates sub-genome-wide significant variants while accounting for horizontal pleiotropy (i.e. effects of the variants through pathways other than the exposure of interest). Correlated SNVs (linkage disequilibrium *R*^2^ < 0.001) were clumped within regions of 10 Mb. Mean *F*-statistics for BW *Z*-score and GA were 10.7 and 10.8, respectively.

Sensitivity analyses used the inverse-variance-weighted, median-based, mode-based, and MR-Egger methods. Additional sensitivity analyses used a more stringent *P*-value threshold (*P* < 5 × 10^−6^) and tested for horizontal pleiotropy using the MR-Egger intercept test. Associations were considered robust if the primary analysis was statistically significant, all sensitivity analyses were directionally consistent, and there was no evidence of horizontal pleiotropy (MR-Egger intercept *P >* 0.05). Due to strong correlation among measures of cardiac and PA structure and function,^8^ we used a false discovery rate *Q*-value < 0.10 to determine statistical significance in primary analyses.

In primary analyses, lower genetically predicted BW *Z*-score was associated with smaller ascending aortic (AA) and right atrial (RA) dimensions, whereas earlier GA was associated with decreased PA, right ventricular (RV), and left atrial size (Figure 1). Sensitivity analyses demonstrated robust associations of lower genetically predicted BW *Z*-score with smaller AA diameter index and RA maximal volume index; earlier GA was robustly associated with smaller systolic PA to AA diameter ratio, RV end-diastolic volume index, and RV stroke volume index (Figure 2). All associations with structural parameters were directionally consistent with estimates using the same parameters not indexed for body size: lower genetically predicted BW *Z*-score was associated with smaller AA diameter [*β* = −0.008 SD (95% confidence interval, CI: −0.013 to −0.002) per 1 SD decrease; *P* = 0.005] and RA maximal volume [*β* = −0.008 SD (95%CI: −0.014 to −0.003); *P* = 0.002], while GA was associated with lower RV end-diastolic volume [*β* = −0.011 SD (95%CI: −0.023–0.000) per 1 SD decrease; *P* = 0.05]. We found no evidence of horizontal pleiotropy (MR-Egger intercept *P >* 0.05 for all). Associations of genetically predicted GA and BW *Z*-score with congenital heart disease and atrial septal defect in FinnGen were non-significant (*P >* 0.05 for all), suggesting that the study findings were not driven by remodelling patterns associated with congenital malformations.

Using summary data from two large, well-phenotyped cohorts, we found that earlier genetically predicted GA was associated with reduced RV and PA sizes, whereas lower BW *Z*-score was associated with smaller RA and AA dimensions. These findings corroborate observational associations for preterm birth and SGA.^1–3^ Our analysis revealed that earlier genetically predicted GA was associated with lower PA and RV dimensions, contrasting with remodelling patterns (PA and ventricular dilation) typically observed in individuals with pulmonary hypertension or heart failure. However, given the increased risk of both pulmonary hypertension and heart failure in preterm-born adults, it is possible that smaller PA and RV dimensions actually contribute to the development of these conditions in such individuals. Smaller ventricular dimensions are associated with impaired functional response to exercise in preterm-born adults,^3^ suggesting that volumetric deficits may contribute to impaired stroke volume reserve in this population. Furthermore, according to Poiseuille’s law, reduced cross-sectional area of the pulmonary vasculature leads to increased PA pressures. By understanding cardiac remodelling patterns associated with early GA and low BW *Z*-score, targeted prevention strategies—including appropriate screening and lifestyle counselling—can be developed to minimize long-term cardiopulmonary consequences. As clinical trials start to evaluate the effects of cardiopulmonary interventions in individuals with high-risk early-life profiles,^10^ screening for preterm birth and SGA may gain clinical actionability in the future.

This study has limitations. First, instrumental variables were constructed using a lenient significance threshold, potentially increasing the risk of weak instrument bias. However, mean *F*-statistics for BW *Z*-score and GA exceeded the conventional weak instrument threshold of 10. Second, our analysis only included European-ancestry cohorts, limiting generalizability to other ancestries. Third, we were unable to evaluate PA pressures, flow metrics, or other functional PA parameters due to unavailability of corresponding genome-wide association data. Finally, our analyses were limited to CMR parameters obtained at rest because genetic analyses on such parameters during exercise are currently unavailable.

This study suggests that lower genetically predicted GA and BW *Z*-score are associated with long-term cardiac and pulmonary vascular remodelling. Our findings underscore the need for targeted prevention strategies to mitigate the burden of early-life risk factors in adulthood.

## Figures and Tables

**Figure 1:**
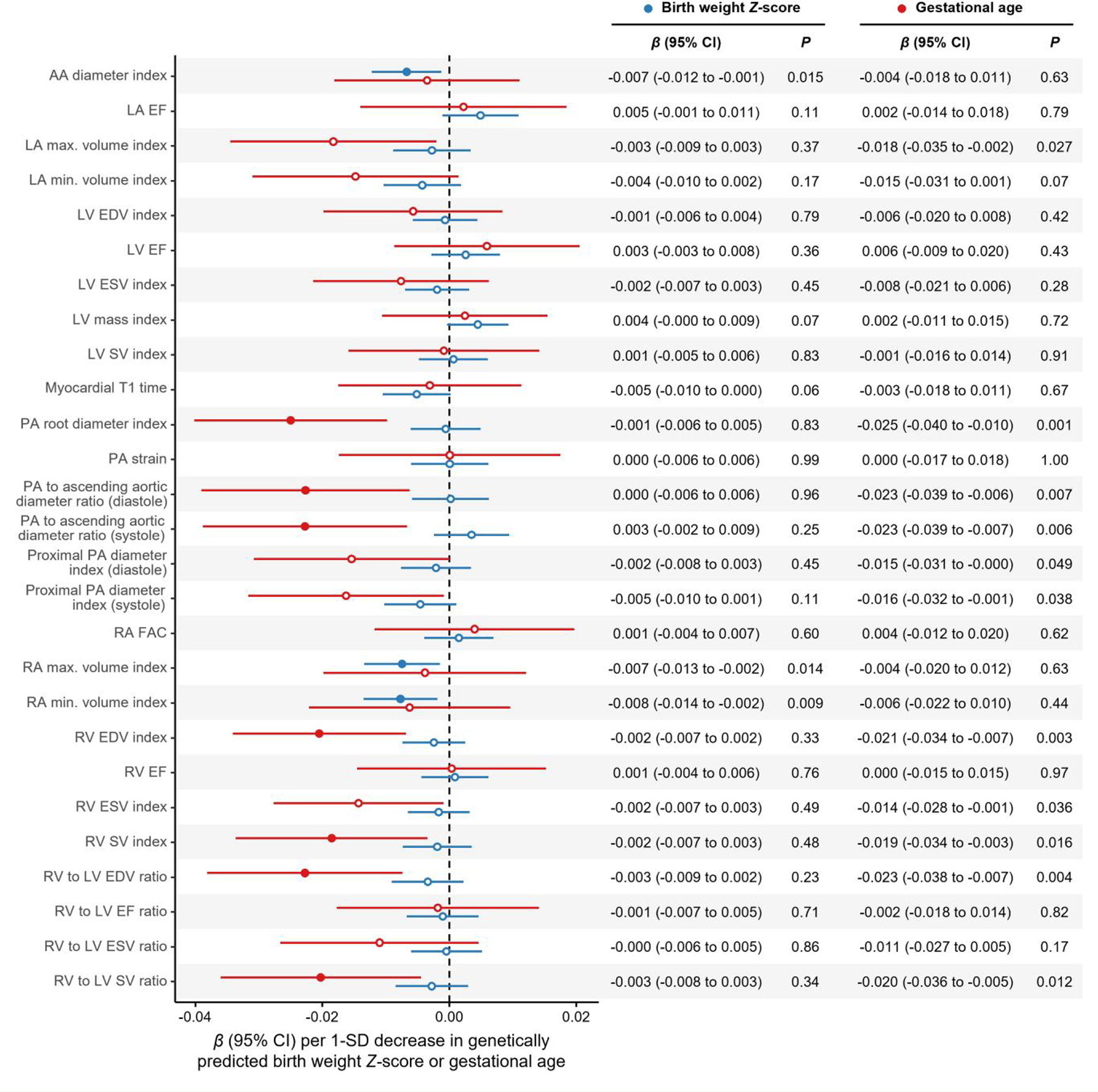
Genetic associations of birth weight *Z*-score and gestational age with measures of cardiac and pulmonary vascular structure and function. Uncorrelated genetic variants associated with birth weight *Z*-score and gestational age (*P* < 5 × 10^−3^ and *R*^2^ < 0.001) were tested as separate exposures, with cardiac magnetic resonance imaging measures of cardiac and pulmonary vascular structure and function as outcomes. Mendelian randomization (MR) was performed using the robust adjusted profile score (MR-RAPS) to accommodate sub-genome-wide significant variants while accounting for pleiotropy. Error bars represent 95% confidence intervals (CIs). Full circles represent associations with *Q*-values < 0.1. All cardiac magnetic resonance imaging parameters are expressed per standard deviation (SD). All associations are expressed per SD decrease in genetically predicted birth weight *Z*-score or gestational age. AA, ascending aorta; EDV, end-diastolic volume; EF, ejection fraction; ESV, end-systolic volume; FAC, fractional area change; LA, left atrial; LV, left ventricular; PA, pulmonary artery; RA, right atrial; RV, right ventricular; SV, stroke volume.

**Figure 2:**
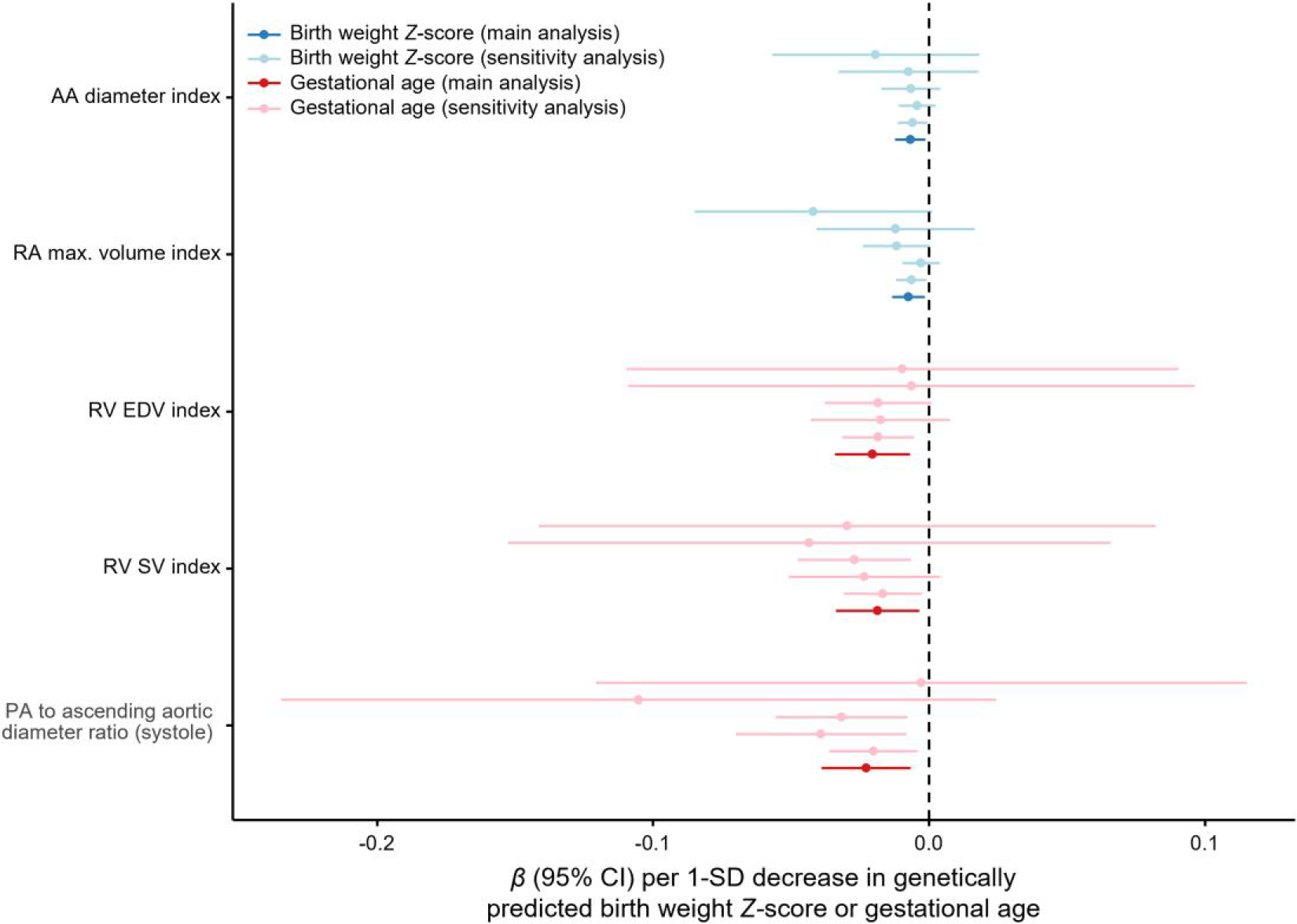
Genetic associations of birth weight *Z*-score and gestational age with measures of cardiac and pulmonary vascular structure and function that were robust to sensitivity analyses. Main analyses included single-nucleotide variants (SNVs) with *P* < 5 × 10^−3^ and the used robust adjusted profile score (MR-RAPS) for causal inference. From top to bottom, sensitivity analyses used (i) MR-RAPS including SNVs at *P* < 5 × 10^−6^; (ii) the simple mode-based method; (iii) the simple median-based method; (iv) MR-Egger; or (v) the inverse-variance-weighted method. Error bars represent 95% confidence intervals (CIs). All associations are expressed per standard deviation (SD) decrease in genetically predicted birth weight *Z*-score or gestational age. AA, ascending aorta; EDV, end-diastolic volume; PA, pulmonary artery; RA, right atrial; RV, right ventricular; SV, stroke volume.

## Data Availability

The data supporting the findings from this study are publicly accessible through the Early Growth Genetics Consortium’s website (http://egg-consortium.org/) and the Cardiovascular Disease Knowledge Portal (https://cvd.hugeamp.org/).
